# Prescription pattern analysis of Type 2 Diabetes Mellitus: a cross-sectional study in Isfahan, Iran

**DOI:** 10.1186/s13040-023-00344-y

**Published:** 2023-10-20

**Authors:** Elnaz Ziad, Somayeh Sadat, Farshad Farzadfar, Mohammad-Reza Malekpour

**Affiliations:** 1 School of Industrial and Systems Engineering, Tarbiat Modares University, Tehran, Islamic Republic of Iran; 2 Centre for Analytics and Artificial Intelligence Engineering, University of Toronto, Toronto, Canada; 3 Non-Communicable Diseases Research Center, Endocrinology and Metabolism Population Sciences Institute, Tehran University of Medical Sciences, Tehran, Islamic Republic of Iran

**Keywords:** Diabetes, Prescription pattern, Association Rule Mining, Multimorbidity

## Abstract

**Background:**

Patients with Type 2 Diabetes Mellitus (T2DM) are at a higher risk of polypharmacy and more susceptible to irrational prescriptions; therefore, pharmacological therapy patterns are important to be monitored. The primary objective of this study was to highlight current prescription patterns in T2DM patients and compare them with existing Standards of Medical Care in Diabetes. The second objective was to analyze whether age and gender affect prescription patterns.

**Method:**

This cross-sectional study was conducted using the Iran Health Insurance Organization (IHIO) prescription database. It was mined by an Association Rule Mining (ARM) technique, FP-Growth, in order to find co-prescribed drugs with anti-diabetic medications. The algorithm was implemented at different levels of the Anatomical Therapeutic Chemical (ATC) classification system, which assigns different codes to drugs based on their anatomy, pharmacological, therapeutic, and chemical properties to provide an in-depth analysis of co-prescription patterns.

**Results:**

Altogether, the prescriptions of 914,652 patients were analyzed, of whom 91,505 were found to have diabetes. According to our results, prescribing Lipid Modifying Agents (C10) (56.3%), Agents Acting on The Renin-Angiotensin System (C09) (48.9%), Antithrombotic Agents (B01) (35.7%), and Beta Blocking Agents (C07) (30.1%) were meaningfully associated with the prescription of Drugs Used in Diabetes. Our study also revealed that female diabetic patients have a higher lift for taking Thyroid Preparations, and the older the patients were, the more they were prone to take neuropathy-related medications. Additionally, the results suggest that there are gender differences in the association between aspirin and diabetes drugs, with the differences becoming less pronounced in old age.

**Conclusions:**

Almost all of the association rules found in this research were clinically meaningful, proving the potential of ARM for co-prescription pattern discovery. Moreover, implementing level-based ARM was effective in detecting difficult-to-spot rules. Additionally, the majority of drugs prescribed by physicians were consistent with the Standards of Medical Care in Diabetes.

**Supplementary Information:**

The online version contains supplementary material available at 10.1186/s13040-023-00344-y.

## Background

The WHO Global Report on Diabetes indicates that the number of Diabetes Mellitus (DM) patients has increased globally in recent decades, from 108 million in 1980 [1] to 537 million in 2021 [2]. This almost fivefold increase was mainly due to the rise in Type 2 Diabetes Mellitus (T2DM) and its risk factors, including obesity, overweight, and ageing [1]. If no proper action is taken to prevent this rise, it has been predicted that 783 million individuals will have diabetes by 2045 (12.2% of the total population) [2]. According to the findings of the national STEPwise Approach to NCD Risk Factor Surveillance (STEPS) 2016, the prevalence of diabetes based on HbA1C in Iran was estimated at 11.0%, 12.7%, and 11.9% for males, females, and both genders, respectively [3].

Regarding the growing epidemic of diabetes [4] and its importance as a global public health problem [5], researchers conducted a variety of in-depth studies to analyze different aspects of this long-lasting disease. Various tools and techniques were employed in diabetes research, the most important of which is data mining. Data mining is used to discover unsuspected relationships, identify patterns, and uncover knowledge from raw data [6]. Some diabetes related applications of data mining methods are as follows: diagnosis and prediction of DM [7], effect of genetic background and environment in the development of diabetes [8], adherence to the clinical guidelines for DM [9], diabetic complications [10], prevention of DM [11], medication recommendation [12], biomarkers identification [13], and patterns of pharmacological therapy [14, 15].

Patterns of pharmacological therapy can be mined by several approaches, one of which is discovering co-prescription patterns to analyze multimorbidity. Multimorbidity, defined as the coexistence of two or more health conditions, poses a significant challenge to public health [16]. Multimorbidity often leads to the associated use of multiple medicines, which is known as polypharmacy [17]. Inappropriate polypharmacy increases the potential for medication nonadherence, drug-drug interactions, and adverse drug events; therefore, analysis of polypharmacy in order to provide valuable insight into current co-prescription patterns has become an interesting discipline [18].

The first objective of this study was to identify the drugs prescribed in combination with Drugs Used in Diabetes for T2DM patients in Isfahan, Iran using their insurance claims data and compare the results with the existing Standards of Medical Care in Diabetes. As a second objective, we investigated the effects of age and gender on prescription patterns. Association Rule Mining (ARM) was the most suitable method for the purpose of this study. ARM is widely used and popular among data mining methods, and aims to find interesting rules from transactional datasets. The main novelty of this study is providing a detailed drill-down analysis of co-prescription patterns for T2DM patients based on different levels defined by the Anatomical Therapeutic Chemical (ATC) coding system.

## Materials and methods

**Fig. 1:**
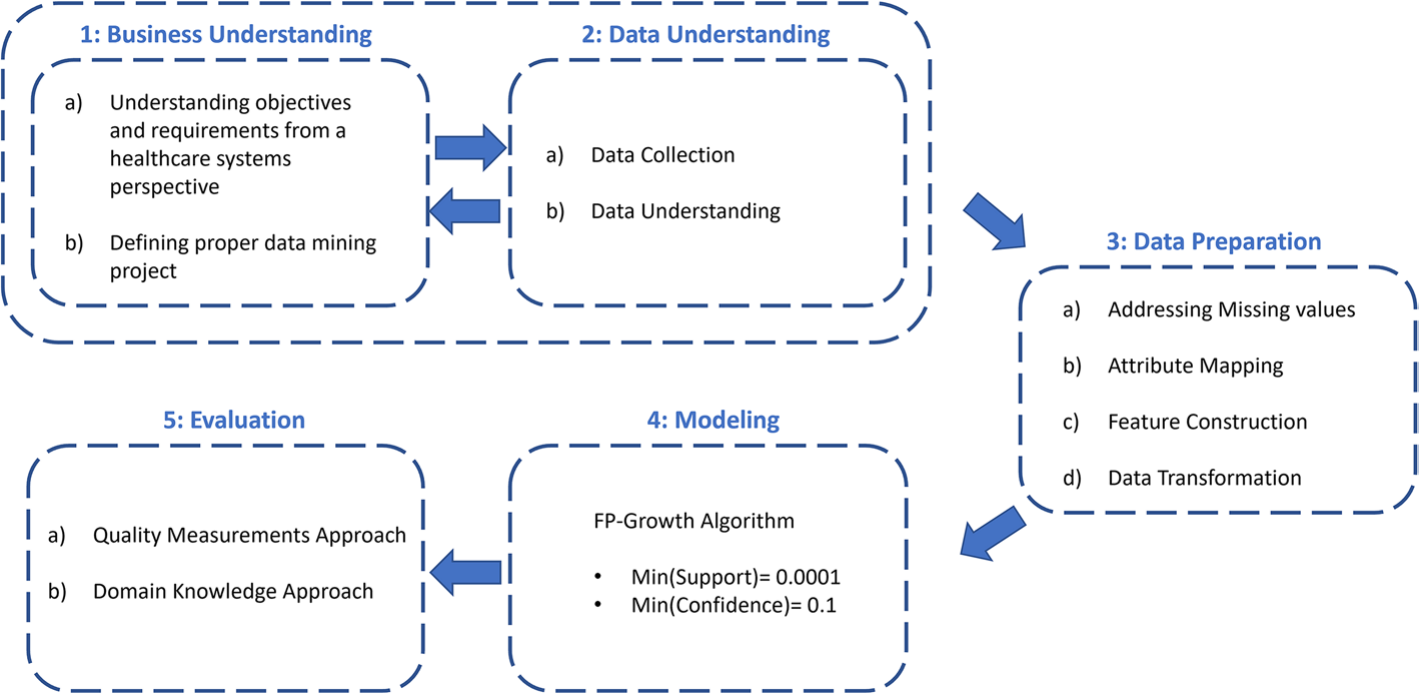
Data analysis pipeline

The data analysis pipeline of this research conforms to the Cross-Industry Standard Process for Data Mining (CRISP-DM) methodology [19] and is depicted in Fig. 1. It contains five phases, including Business Understanding, Data Understanding, Data Preparation, Modeling, and Evaluation.

### Phase 1 and 2: business and data understanding

There is a close link between phase 1 and phase 2 since formulating the data mining problem requires at least some understanding of the available data.

The datasets used in this study were acquired from the Iran Health Insurance Organization (IHIO). The following tables were retrieved from the IHIO data warehouse:

1. Electronic insurance claims fact table, which contained fields for drug prescription information, patient demographics, and the prescriber’s medical council’s code. This table contained the prescriptions of 914,652 unique patients.
2. Dimension tables, first of which contained national drug codes as keys and different levels of ATC drug codes as values, and the second of which had the medical council’s codes as key and the specialty of the physicians as values.

We also needed to understand the study requirements and objectives from a healthcare systems perspective, as well as define a proper data mining project to achieve research objectives.

Insurance claims data tend to be incomplete, and one of the major requirements for any prescription-based study is the diagnosis label. The data used in this study lack the International Classification of Disease (ICD) code for each prescription, which is why the first challenge is to answer this question: “How to identify patients with T2DM using insurance claims data accurately?” Therefore, a rule framework was implemented based on some clinical assumptions to differentiate between T2DM patients taking drugs from the A10 subgroup and patients taking them for other reasons such as Polycystic Ovary Syndrome (PCOS), Type 1 Diabetes Mellitus (T1DM), or pregnancy. A panel of five medical experts defined the following criteria to recognize T2DM in subjects:

1. Presence of antidiabetic drugs in the prescription:
  - Oral Anti Diabetic agents (OADs)
  - Insulin

Antidiabetic drugs can be determined by means of the ATC classification system. In this system, the active substances are divided into different groups according to the organ or system on which they act and their therapeutic, pharmacological, and chemical properties [20]. A10 is a predefined level of ATC, indicating Drugs Used in Diabetes. If a prescription contains at least one of the A10 drugs, there is a possibility that the patient suffers from T2DM. However, it is not the sole criterion for labeling T2DM patients.

2. The specialty of prescribing physician:
  - General practitioner
  - Internist
  - Endocrinologist

If the prescriber’s specialty is one of the mentioned specialties, it raises the possibility that the prescription belongs to a T2DM patient.

3. Checking for other conditions
  - T1DM in males (if the prescription belongs to a male patient under the age of 30 and contains any kind of Insulin)
  - T1DM in females (if the prescription belongs to a female patient under the age of 30 and contains any kind of Insulin except Insulin Human)
  - PCOS (if the prescription belongs to a female patient under the age of 30 and contains metformin prescribed by a gynecology physician)
  - Pregnancy (if the prescription is for a female patient under the age of 30, contains Insulin Human, and the patient is not on the list of patients who have taken at least one OAD in a non-PCOS prescription)

Subjects who met the criteria in previous steps may also have T1DM, PCOS, or be pregnant. Therefore, it is crucial to draw a distinction between T2DM and other conditions.

To sum up, when a patient takes one of the A10 subgroup drugs (excluding Insulin siring), the prescriber has one of the mentioned specialties, and the patient does not suffer from other conditions, the patient can be labeled as a T2DM patient. In this study, 91,505 patients were labeled as having T2DM, and A10 drugs were removed from the prescriptions of patients who were not labeled as having T2DM.

To convert the objective of this study to a data mining problem definition, the ARM algorithm was selected in this phase. Several ARM algorithms were developed, including Apriori, Eclat, and FP-Growth. In this study, FP-growth was selected because it is faster, more efficient, more scalable than other algorithms, and it is used in recently published articles [21, 22].

### Phase 3: data preprocessing

Apache Spark [23], version 3.2.0, a unified big data processing engine, was utilized for data preprocessing. A brief description of this procedure is provided in the subsections below.

**Fig. 2:**
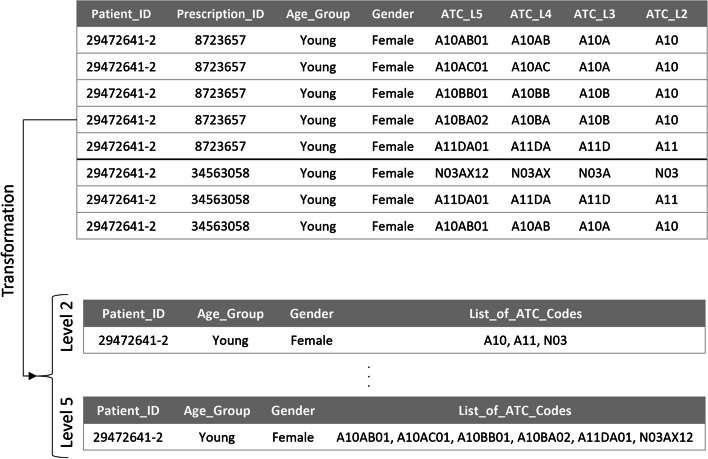
Data transformation

- **Addressing missing values**
  The patients with missing gender and age are just a random subset of all patients, so there are no meaningful differences between these patients and the others. It implies a Missing Completely At Random (MCAR) situation, in which it is safe to remove rows with missing values because the results will be unbiased. Therefore, missing values were handled by deleting the entire prescriptions of patients having NULL gender and age.
- **Attribute mapping**
  Attribute mapping is the act of transforming and/or connecting one or more attributes to a new attribute or set of attributes. In this step, attribute mapping was used as a solution to the following problem:
  Drug codes were based on the national Food and Drug Administration coding system in the insurance claims dataset. It was necessary to transform national drug codes into international ones that are based on the ATC Classification System. First, a dataset that contained the corresponding ATC codes for every national drug code was retrieved. Then, attribute mapping was carried out to retrieve different levels of ATC codes in separate columns (Table 1). The same action was taken to map the medical counsel’s code of prescribers to their specialty and the age of the patient to the matching age category (Tables 2 and 3). In this study, three age categories were defined as follows:
  - ◦ Age < 30 -> Young
  - ◦ 30 <= Age < 45 -> Middle-Aged
  - ◦ Age >= 45 -> Old
- **Feature construction**
  Patients’ national codes, which were unquestionably unique for each individual, were not available due to data anonymization. Therefore, creating a unique ID for each patient was necessary. In the available dataset, Insurance_ID was not unique. Family members had the same Insurance_ID but different Insurance_Serials. To create a unique ID for each patient, the Insurance_ID and Insurance_Serial features were combined and named Patient_ID.
- **Data transformation**
  This step involved data transformation so it could be used as input for the FP-Growth algorithm. First, the data were aggregated at the patient level for each level of ATC. Second, duplicate ATC codes were removed to create a unique list. This procedure was done for all combinations of age categories and genders. In Fig. 2, an example of this transformation is provided.

**Table 1:** Attribute mapping for national drug ID

…	National_Drug_ID	Generic_Drug_Name	ATC_L5	ATC_L4	ATC_L3	ATC_L2	ATC_L1
…	02023	Asa 100 mg - tablet	B01AC06	B01AC	B01A	B01	B

**Table 2:** Attribute mapping for prescriber medical council code

…	Prescriber_Medical_Counsil_Code	Prescriber Specialty
…	1787	Endocrinologist

**Table 3:** Attributing mapping for age

…	Age	Age_Category
…	38	Middle-Aged

### Phase 4: modeling

FP-growth is currently one of the fastest algorithms for finding frequent patterns in transactional databases due to its independence from candidate generation and ability to store a compact version of the database in memory [21]. In the first step, this algorithm finds frequent items by calculating their frequencies. In the second step, it uses a suffix tree (FP-tree) structure to encode transactions without explicitly generating candidate sets. Finally, the frequent itemsets can be extracted from the FP-tree. In this study, FP-growth was implemented in Apache Spark [23] using the FPGrowthModel.

Another indicator, named prevalence, was also computed for the extracted rules of the second level of ATC. Let us denote Drugs Used in Diabetes as A and another drug as B. Regarding the formula for support, Supp(A → B) = σ(A → B)/N, and knowing the value of N, 914,652, we can calculate σ(A → B), which shows the number of patients who took drugs A and B. In this section, we calculated the proportion of σ(A → B) to only diabetic patients, 91,505, to compute the prevalence of prescribing drug B among T2DM patients.

### Phase 5: evaluation

In this phase, the details of the utilized validation approaches will be explained.

- **Quality measurements approach:** Not all the rules generated by ARM algorithms are meaningful. Quality measurements can be helpful in excluding some coincidental rules. Each Association Rule (AR) has two primary quality measurements, namely support and confidence, defined as:
  **Support criterion:** the support of an AR denoted by Supp(A → B) is defined as Supp(A → B) = σ(A → B)/N, where σ(A → B) is the number of patients who have taken drugs A and B, and N is the total number of patients. In sum, support is simply the frequency of occurrence of each rule.
  **Confidence criterion:** the confidence of an AR denoted by Conf(A → B) is defined as Conf(A → B) = (Supp(A → B))/(Supp(A)), where Supp(A) is the number of patients who have taken drug A. In summary, confidence is the strength of implication.

A rule is considered valid if it satisfies the below conditions:

- Support(A → B) ≥ Minisupp
- Confidence(A → B) ≥ Miniconf

In this study, we performed a grid search and opted for support = 0.0001 and confidence = 0.1 to uncover valuable associations across different patient segments while avoiding redundancy.

b) **Domain knowledge approach**: after passing the previous step, two medical doctors investigated the remaining rules in order to check clinical rationality and possible indications.

## Results

Overall, we analyzed the prescriptions of 914,652 unique patients, of whom 91,505 were diabetic. It suggests that the prevalence of T2DM in our sample was approximately 10%. In total population, there were 510,873 female and 403,779 male patients; these figures were 58,023 and 33,482 for diabetic patients. Regarding age groups, there were 355,925 patients in the “young” category, followed by 203,166 and 355,561 in the “middle-aged” and “old” categories. The mentioned figures were 6,750, 14,092, and 70,663 in the diabetic population, respectively. The average age of the total population was 38.67, with a standard deviation of 21.78, while diabetic patients were on average 56.08 years old, with a standard deviation of 15.83. In the following subsections, we decided to present the top 30 rules of each ATC level with the condition of lift value above 2.0; therefore, some levels had less than 30 ARs regarding this criterion.

### Second level of ATC

ARs were sorted by prevalence indicator since we wanted to present the most prescribed drug classes (Table 4). According to this indicator, Lipid Modifying Agents (C10) with 56.3%, Agents Acting on The Renin-Angiotensin System (C09) with 48.9%, Antithrombotic Agents (B01) with 35.7%, and Beta Blocking Agents (C07) with 30.1% were the most prevalent drug classes prescribed with Drugs Used in Diabetes (A10).

**Table 4:**
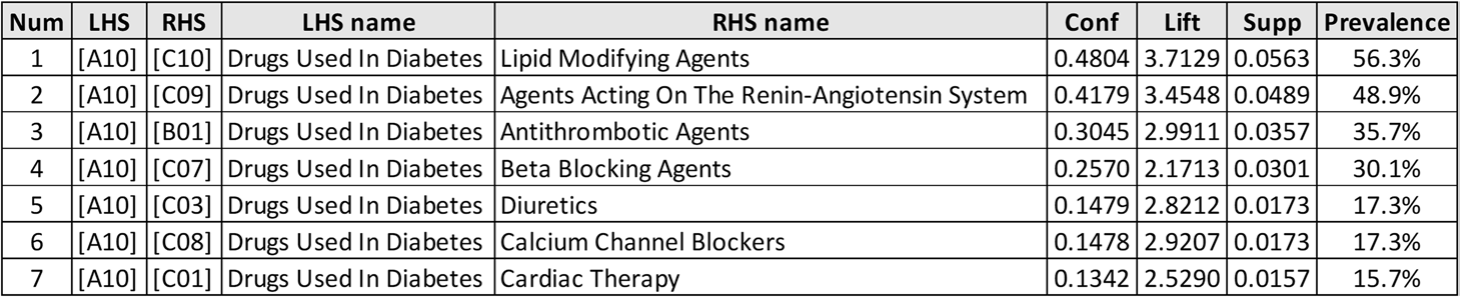
ARs of the 2^nd^ ATC level

### Third level of ATC

Patients who took both Insulins and Analogues (A10A) and Blood Glucose Lowering Drugs, Excl. Insulins (A10B) were more likely to take ACE Inhibitors, Plain (C09A) by 5.43 times, followed by Vitamin B1, Plain and In Combination with Vitamin B6 And B12 (A11D; 5.39), Angiotensin II Receptor Blockers (ARBs), Plain (C09C; 5.08), Lipid Modifying Agents, Plain (C10A; 4.87), and Antithrombotic Agents (B01A; 4.17) (Table 5).

**Table 5:**
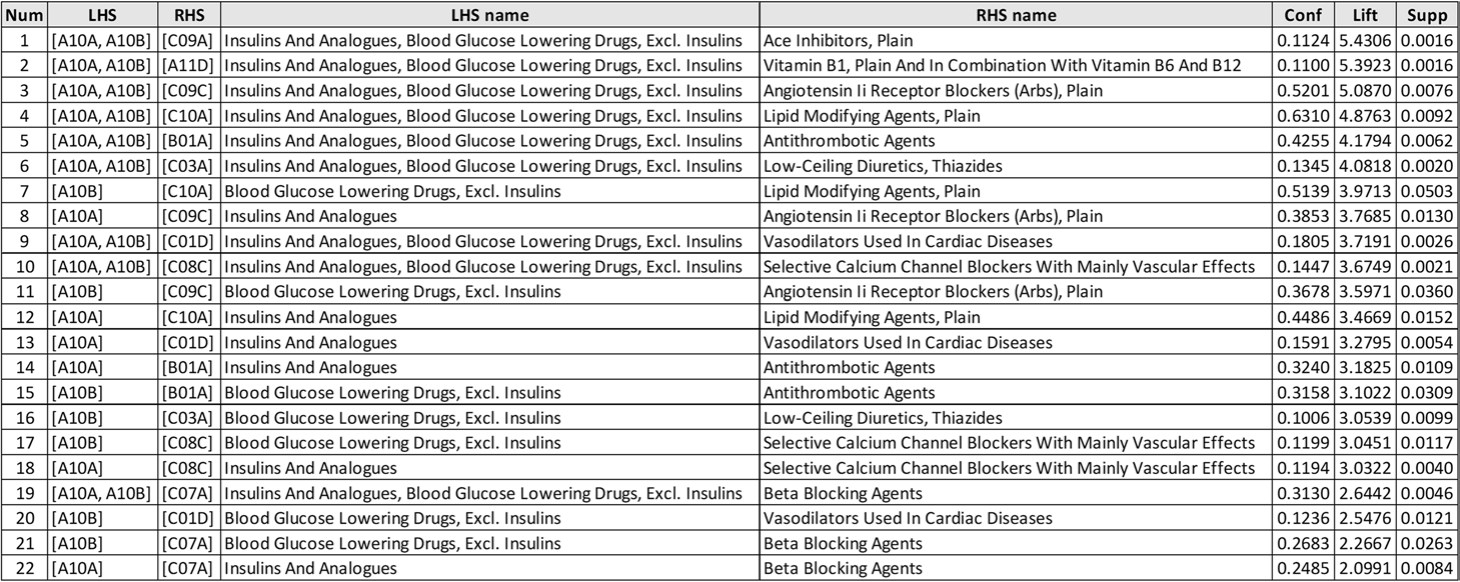
ARs of the 3^rd^ ATC level

### Fourth level of ATC

The top rule regarding lift value was “Insulins and Analogues for Injection, Intermediate-Acting, Biguanides, Sulfonylureas =  > Ace Inhibitors, Plain”, with a lift of 10.13 (Table 6). Another meaningful rule had the same LHS as the previously mentioned top rule, and its RHS is Vitamin B1, Plain, with a lift of 9.04. The ARs number 7, 9, 14, 19, and 20 suggest that among C03C, C03A, C01D, C10A, and B01A subsets, Sulfonamides, Plain (C03CA), Thiazides, Plain (C03AA), Organic Nitrates (C01DA), HMG-CoA Reductase Inhibitors (C10AA), and Platelet Aggregation Inhibitors Excl. Heparin (B01AA) were the main choices of physicians, with lift values of 7.66, 6.59, 6.28, 6.28, and 6.06, respectively.

**Table 6:**
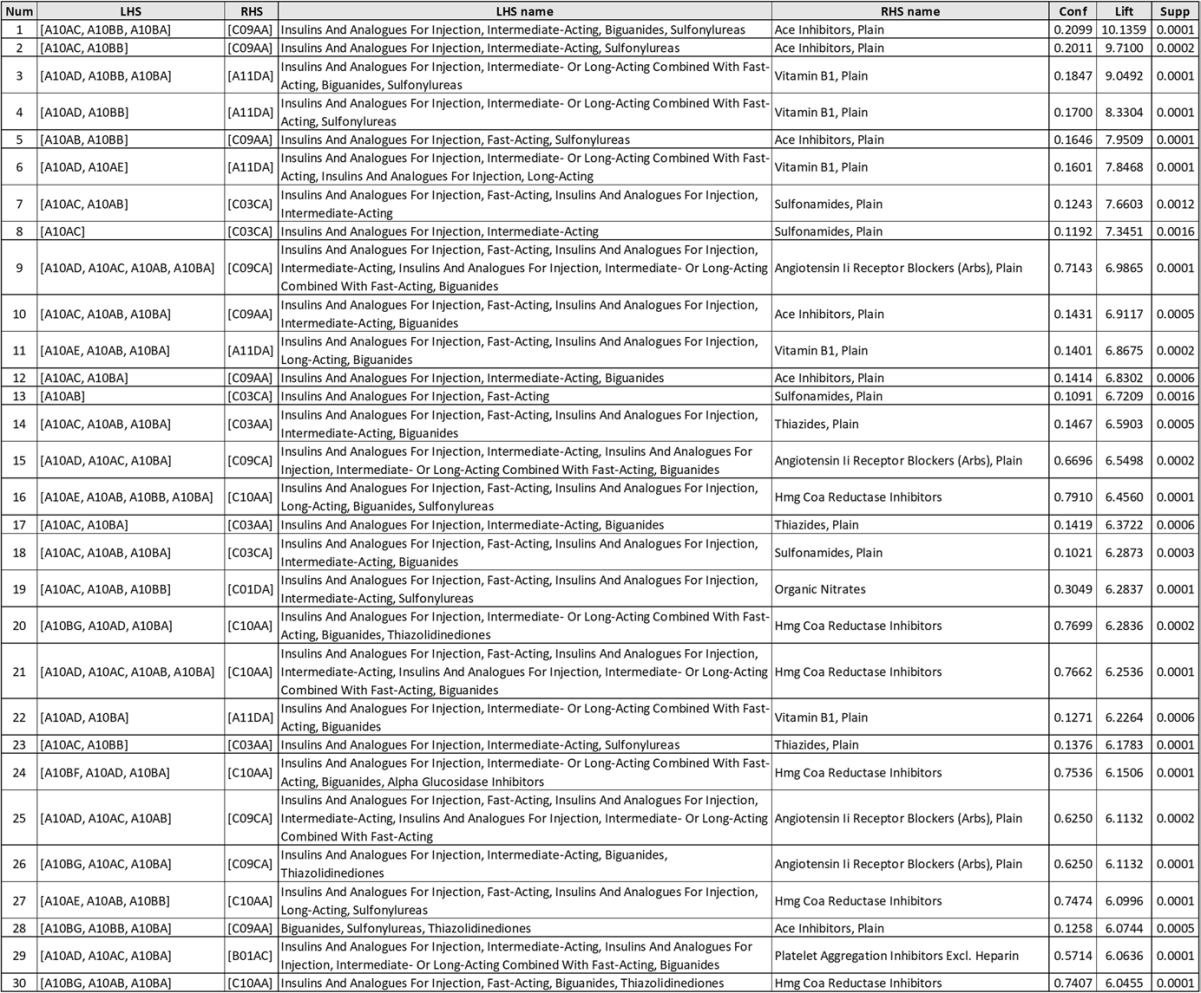
ARs of the 4^th^ ATC level

### Fifth level of ATC

According to the results of this level, “Insulin Aspart, Glibenclamide =  > Thiamine (Vit B1)” and “Insulin (Human) =  > Furosemide” had the highest lift value, with 9.35 and 8.08, respectively (Table 7). Regarding the RHS, ARs associated with Losartan, Atorvastatin, Hydrochlorothiazide, and Gabapentin had the highest lift values, including “Glibenclamide, Gliclazide, Pioglitazone =  > Losartan” with a lift of 7.08, “Insulin Aspart, Metformin, Pioglitazone =  > Atorvastatin” with a lift of 6.66, “Insulin (Human), Insulin (Human), Metformin =  > Hydrochlorothiazide” with a lift of 6.61, and “Insulin Aspart, Insulin Glargine =  > Gabapentin” with a lift of 6.44 (Table 7).

**Table 7:**
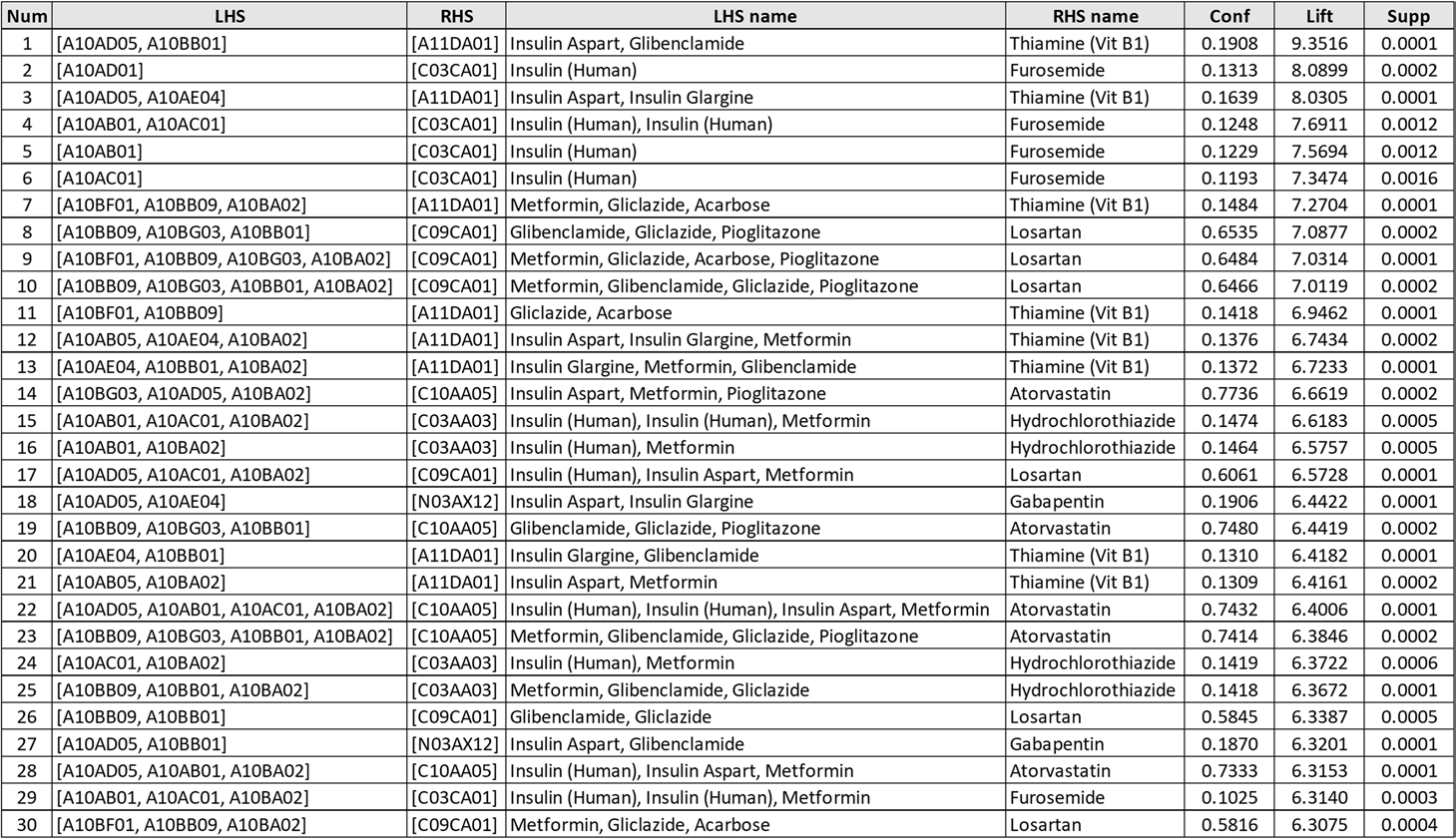
ARs of the 5^th^ ATC level

### Age and gender-wise results

The ARs extracted for each combination of gender and age groups are presented in an additional file (see Additional file 1).

## Discussion

### Second level of ATC

Although it is not accurate and straightforward to imply the exact prevalence of diabetes complications from the percentage of prescribed drugs, Table 4 may at least approximately suggest the prevalence order of the main diabetes complications. According to medical experts’ opinions, C10, C09, and B01, which are the top three associated medications, are mainly prescribed for treating Dyslipidemia, Hypertension, and Cardiovascular disease, respectively.

### Third level of ATC

The results of ARM analysis of the third level of ATC (Table 5) showed that ARs with Right-Hand-Side (RHS) of ACE Inhibitors (C09A) or Angiotensin II Receptor Blockers (ARBs) (C09C) had significant values for the lift indicator. There is a clear recommendation in the Standards of Medical Care in Diabetes - 2021 [24] about the treatment of Hypertension in DM patients. Diabetic patients’ treatment of Hypertension should include drug classes that reduce Cardiovascular (CV) events in patients with diabetes. C09A or C09C are recommended as first-line therapy for Hypertension in individuals with diabetes and Coronary Artery Disease (CAD). Therefore, the mentioned co-prescription is aligned with diabetes treatment guidelines.

### Fourth level of ATC

First, as shown in the results, there is a strong AR indicating the prescription of HMG CoA Reductase Inhibitors (C10AA) with Drugs Used in Diabetes. According to the Standards of Medical Care in Diabetes - 2021 [24], T2DM patients have an increased prevalence of Lipid Abnormalities, contributing to their high risk of Atherosclerotic Cardiovascular Disease (ASCVD). Statin therapy has proven effects on ASCVD consequences in subjects with and without Congenital Heart Disease (CHD). Statins are the drugs of choice for Low-Density Lipoprotein (LDL) Cholesterol-lowering and Cardioprotection for both primary and secondary prevention. Therefore, the above-mentioned AR suggests that physicians adhere to the treatment guidelines.

Second, according to our results, the prescription of Platelet Aggregation Inhibitors excl. Heparin (B01AC) is meaningfully associated with Drugs Used in Diabetes. In fact, it is proved that diabetic patients are more likely to develop Coronary and Peripheral Vascular diseases than non-diabetic subjects, and B01AC is known for the primary and secondary prevention of vascular events [18]. That is the reason why the prescription of B01AC is common in these patients. Therefore, our result is aligned with the diabetes treatment approach.

Third, the results of the ARM analysis of the fourth level of ATC showed that the prescription of Organic Nitrates (C01DA) is highly associated with Drugs Used in Diabetes. Since diabetes-associated vascular dysfunction, as well as Nitrate Tolerance, adequately responds to antioxidant therapy, this may indicate the efficacy of prescribing Organic Nitrates in diabetic patients [25].

### Fifth level of ATC

First, among Angiotensin II Receptor Blockers (ARBs), Plain (C09C) drugs, Losartan (C09CA01) as an RHS had the most significant lift. Although the main effects of Losartan are due to its ATC class and are the same as those of other approved ARBs, it also has some unique benefits. For instance, a shorter duration of action, uricosuric effect, attenuation of platelet aggregation, and protective effect on the kidney [26]. Our results suggest that physicians preferred prescribing an ARB with some effects on other diabetes complications.

Second, Hydrochlorothiazide (C03AA03) and Furosemide (C03CA01) are not commonly prescribed for controlling high blood pressure. However, the results show strong lift values for these drugs. It is mainly due to their Renoprotective [27] and Cardioprotective [28] effects, which are needed to treat common diabetes complications such as Renal Insufficiency and Heart Failure.

Third, the extracted ARs of the fifth level of ATC indicate that Thiamine (vit B1) (A11DA01) is more likely to be prescribed to diabetic than non-diabetic patients. The clinical effectiveness of Thiamine (vit B1) in controlling diabetic complications has been investigated in several studies [29, 30]. According to their results, this vitamin has an effective role in Diabetic Endothelial Vascular Diseases, Lipid Profile, Nephropathy, Cardiopathy, Retinopathy, and Neuropathy.

Fourth, the findings of co-prescription patterns in the fifth level of ATC have evidenced the strong bond between Gabapentin (N03AX12) and Drugs used in diabetes. Although Gabapentin is primarily used as an anti-epileptic agent, there is an increasing pattern of its utilization as an initial pharmacologic treatment in diabetic neuropathy [31]. The added value of the hierarchical analysis of co-prescription patterns performed in this study was finding such strong rules hidden throughout previous steps.

Fifth, another interesting finding is that Thiamine (vit B1) and Gabapentin are usually associated with the prescription of Insulin which is a sign of more advanced stages of diabetes.

### Age- and gender-wise findings

The most noticeable difference found in the gender-wise analysis was the higher lift value of Thyroid Preparations in females compared to males. Although this finding is sporadically mentioned in the literature, the necessity of thyroid function screening is not well established in the clinical guideline of diabetes management [24, 32, 33].

Furthermore, our results revealed that Acetylsalicylic Acid (Aspirin) was found to be associated with some of the Drugs Used in Diabetes in the young age category of males, whereas this association was not observed in young females. This gender difference was also evident in the middle-aged group, where the number of extracted ARs for females was almost half that of males, and the average support, confidence, and lift for middle-aged males were higher than those for females. However, in the old age category, the number of rules for females increased and even slightly exceeded that for males, and we observed slight differences in quality measurements of the mentioned AR for males and females. This may suggest that the gender differences in the association between Aspirin and Drugs Used in Diabetes become less pronounced as individuals age.

In age-wise analysis, the results revealed that as one gets older, Vit B1 appears in the top rules, which may be an indication of neuropathy onset.

### Overall findings and possible implications

Through data mining, we revealed the current pattern of diabetes management in a developing country, and showed that the care provided in Iran is relatively evidence-based and physicians mostly adhere to guidelines. This is an important finding, as it suggests that the healthcare system in Iran is providing appropriate diabetes care despite limited resources. Additionally, we identified the order of most frequent comorbidities associated with diabetes, which can inform care by providing healthcare decision-makers and strategists with valuable insights to prioritize their efforts and resources towards the most prevalent comorbidities. This may lead to more effective management of diabetes. Furthermore, based on our findings, we would like to suggest that diabetes guidelines consider including thyroid function screening, particularly for females with diabetes. It seems to be crucial for physicians to consider lab tests of thyroid disorders in this population, as this could have significant implications for preventive care and diabetes management.

### Strengths and limitations

The results are based on data from the total rather than a sample of insured patients in Isfahan city; therefore, the risk of selection bias is low. To the best of our knowledge, previous similar studies were conducted with roughly a thousand samples [34–39]. In this study, we examined the prescriptions of 91,505 diabetic patients, which is way beyond the sample size of other research projects in this discipline. Additionally, the mentioned studies only found the prescription patterns regarding one level of ATC. However, we drilled down into all ATC levels for a comprehensive analysis.

Since our database is comprised of claims data, all patients were insured, and the results might not apply to the general population. In addition, some insured patients would have preferred to purchase their medications over-the-counter (OTC), and therefore no medical prescription was captured for them. Moreover, the lack of ICD-Code was one of the main limitations of this study, and this challenge was met by implementing a rule framework based on medical experts’ opinions.

## Conclusions

The analysis carried out in this paper showed (I) the potential of association rules for pattern discovery and mining of healthcare databases because the majority of the ARs were clinically meaningful, (II) physicians’ drugs of choice were mostly aligned with the Standards of Medical Care in Diabetes - 2021 [24], (III) the capability of insurance claims database as a proxy for clinical diagnoses, (IV) the effectiveness of implementing level-based ARM to find meaningful rules which were difficult to spot.

### Supplementary Information

**Additional file 1.**

## Data Availability

The data used in this study is owned by IHIO. Therefore, authors are not allowed to share the data publicly or privately. However, any researcher with written permission from IHIO can request to obtain the anonymized data.

## Abbreviations

AR: Association Rule
ARM: Association Rule Mining
ASCVD: Atherosclerotic Cardiovascular Disease
ATC: Anatomical Therapeutic Chemical
CAD: Coronary Artery Disease
CHD: Congenital Heart Disease
CRISP-DM: Cross-Industry Standard Process for Data Mining
DM: Diabetes Mellitus
CV: Cardiovascular
ICD: International Classification of Disease
IHIO: Iran Health Insurance Organization
LDL: Low-Density Lipoprotein
LHS: Left-Hand-Side
MCAR: Missing Completely At Random
OADs: Oral Anti Diabetic agents
OTC: Over-The-Counter
PCOS: Polycystic Ovary Syndrome
RHS: Right-Hand-Side
STEPS: STEPwise Approach to NCD Risk Factor Surveillance
T1DM: Type 1 Diabetes Mellitus
T2DM: Type 2 Diabetes Mellitus
